# An integrated georeferenced dataset of public investments for soil defence in Italy

**DOI:** 10.1016/j.dib.2024.110909

**Published:** 2024-09-05

**Authors:** Lorena Ricciotti, Alessio Pollice

**Affiliations:** Università degli Studi di Bari Aldo Moro, Largo Abbazia Santa Scolastica, 70124 Bari, Italy

**Keywords:** Public works, Financial data, Spatial Data, Italian municipalities, Record linkage

## Abstract

The dataset collects and harmonizes financial data about public works in Italy, focusing on soil defence investments. The data are sourced from three distinct platforms: the Italian Ministry of Economics and Finance's open data platform OpenBDAP, the OpenCoesione website, concerned with interventions framed in cohesion policies financed by additional resources from the European and national budgets, and the ReNDiS database, provided by the Italian Institute for Environmental Protection and Research (ISPRA), that exclusively gathers information about public works in soil defence. The data records belonging to these three sources are linked by a unique project code (CUP), ensuring that there is no duplication of data. The OpenBDAP and OpenCoesione repositories report financial variables classified into various funds. In contrast, the ReNDiS database only provides the total amount of financial resources allocated to each intervention. Consequently, the merged dataset consolidates these financial variables into one, representing the total investment amount. For the first two databases this aggregate is derived by summing the financial flows from the different funds. Geographical referencing has been added to each intervention and each financial observation is associated with an Italian municipality. The database includes information on the region, province, and municipality for each record. Each database entry has also been equipped with the coordinates of the municipality's centroid and with the polygonal shape of the municipality area. Overall, the merged dataset encompasses 28 variables reporting three descriptive variables, one financial variable representing the total amount of financial resources, six geographic variables representing the codes and names of regions, provinces, and municipalities, sixteen variables referring to key dates of the process of public works, two geographical references variables respectively representing the centroids and the shape polygons of the municipality. This comprehensive dataset allows to analyse the spatial distribution of the resources allocated to soil defence investments. It offers insights to policymakers striving to allocate resources more efficiently, thereby fostering sustainable land management practices and ensuring the long-term health of the Italian ecosystems. The dataset can be complemented with additional information related to various concomitant aspects such as those pertaining to the environmental and socio-economic fields. This integration allows for broad analysis of the relationships between soil defence efforts and surrounding environmental and socio-economic contexts.

Specifications Table

**Table:** 

Subject	Economics, Econometrics and Finance: Climate and Environmental Finance
Specific subject area	Geo-spatial information on soil defence investments for efficient resource allocation and policy formulation.
Type of data	Table, CSV. Raw, Filtered, Processed, Geodata
Data collection	Data were collected from three different repositories: OpenBDAP, OpenCoesione, and ReNDiS. The RStudio software was used for the whole process of data retrieving, harmonization and geo-referencing. Data were downloaded by SPARQL queries and API's and then filtered for soil defence projects. The three datasets were merged avoiding duplication and ensuring data coherence. To avoid duplication the Unique Project Code was used for record linkage. Geographical references were added to each record using ISTAT codes referred to regions, provinces, and municipalities. Thanks to this references, geo-referencing was added at municipal level with both the centroid coordinates and the polygon shapes of each municipal area.
Data source location	Country: Italy 1. Institution: Dipartimento per le politiche di coesione e per il sud - Presidenza del Consiglio dei Ministri – Italian Government Primary data source: OpenCoesione 2. Institution: Ministero dell'Economia e delle Finanze – Italian Government Primary data source: OpenBDAP 3. Institution: Istituto Superiore per la Protezione e la Ricerca Ambientale (ISPRA) Primary data source: ReNDiS
Data accessibility	Repository name: Zenodo and CRAN Data identification number: 10.5281/zenodo.13190302 Direct URL to data (Zenodo): https://zenodo.org/records/13190302 Zenodo is a platform provided by the CERN and OpenAIRE that allows researchers to upload and share their data. Direct URL to data (CRAN): https://cran.r-project.org/web/packages/PublicWorksFinanceIT/index.html CRAN is a network of ftp and web servers around the world that store identical, up-to-date, versions of code and documentation for R.

## 1 Value of the Data

• The dataset on public works for soil defense in Italy is the result of merging the data from three different repositories: OpenBDAP, OpenCoesione and ReNDiS. This merging process enhances the richness and completeness of the dataset providing a comprehensive overview of soil defense financial efforts across the country. By consolidating information from multiple sources, the dataset becomes valuable for policymakers and researchers alike, offering a unified and comprehensive resource for analysis and decision-making.
• The data holds significant value for various stakeholders, especially Italian policymakers and researchers, due to its specificity. This specificity is crucial as the data provides detailed information for each individual project and can be aggregated at the municipality level. The dataset's uniqueness lies in its comprehensive coverage of each municipality, representing a notable advancement in soil defense research. Unlike previous studies that often-lacked granularity, this dataset delves into localized investment details. As a result, it enables deep insights into investments made and outcomes achieved, supporting informed decision-making, resource allocation, and the development of innovative solutions to mitigate soil-related risks. Additionally, the availability of such detailed and comprehensive data promotes collaboration and knowledge-sharing among stakeholders involved in soil defense.
• This dataset on soil defense investments in Italy, incorporates also the temporal dimension. Through temporal analysis, researchers can track trends in funding allocations and policy emphasis over time, providing insights into the evolution of soil defense strategies and priorities. This temporal approach allows for the identification of critical periods of investment growth or decline, highlighting shifts in governmental priorities or environmental concerns. Moreover, researchers can assess the long-term effectiveness of soil defense initiatives by examining how investment patterns correlate with changes in soil consumption indicators over time. By leveraging this temporal perspective, researchers can not only understand current investment trends but also anticipate future needs and challenges, enabling proactive decision-making to safeguard Italy's soil resources for generations to come.
• This dataset can seamlessly integrate with other datasets on various levels. For instance, it can be coupled with environmental data to explore how environmental factors influence the allocation of funding for soil defense public works. Additionally, socio-economic indices can be utilized to analyze whether contextual factors impact expenditure patterns. This interoperability enables researchers and policymakers to conduct comprehensive analyses, uncovering correlations and insights that can inform more effective decision-making and resource allocation strategies in soil defense initiatives.

## 2 Background

Public works encompass infrastructures and services that are typically financed and undertaken by government authorities to serve the collective needs of the society. They relate to a diverse range of facilities and initiatives, including transportation systems, water and sewage treatment facilities, energy infrastructure, public buildings, and other essential structures. These are designed to enhance the overall well-being of the community by fostering economic development, ensuring public safety, and improving the quality of life for residents. Particularly, public works for soil defence mitigate the hydro-geological risks posed by various environmental factors, such as erosion, landslides, and flooding. They aim at safeguarding both infrastructures and ecosystems. The motivation behind constructing a dataset of public works for soil defence stems from the necessity to address some key challenges in the research on public policies for environmental management. Existing datasets often lack consistency and specificity, impeding researchers' ability to analyse trends and assess the effectiveness of public investments comprehensively [4]. As the digital landscape evolves, there's a growing demand for comprehensive, organized data to facilitate evidence-based decision-making [2,3,5,10]. By aggregating detailed information on soil defence investments across Italy, including temporal and spatial dimensions, this dataset fills a critical gap, offering researchers a nuanced understanding of evolving funding patterns and policy priorities.

## 3 Data Description

**Fig. 1:**
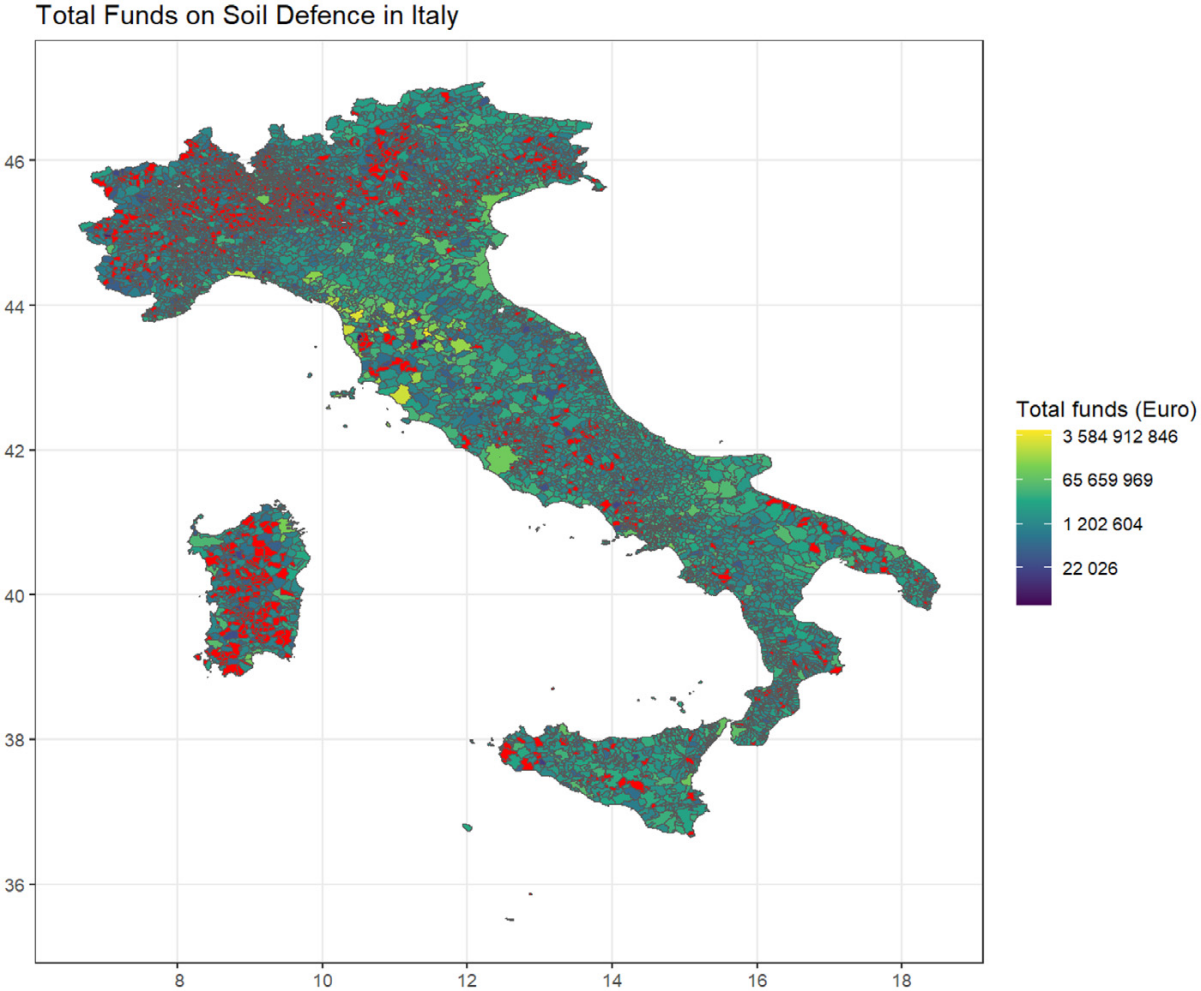
Italy map on soil defence total investment. Red areas show municipalities without information on soil defence investments.

The dataset provides comprehensive information on soil defense public works conducted throughout Italy (Fig. 1). Specifically, it includes details on the total funding allocated to each intervention, along with precise location data indicating the municipality where each project was implemented. The dataset is available on Zenodo [1], and its latest static version is updated as of February 2024. This particular version was created on March 16, 2024, at 4:50 PM. It is composed of four different folders named after the sources from where data are taken plus one called “Data Region”, where the merged version of the three datasets can be found. Each folder contains: datasets for each Italian region, hence 20 files. Names of each dataset are composed of "sd_" which stands for "soil defense", followed by the name of the source, thus "obdap", "oc" or "rendis, followed by _regionname. In addition, the main folder includes a file called "Italy.csv" which contains the data on soil defense for the entire country, and a file named "metadata" where names of the variables and information on them can be found. Available datasets are in CSV format and contain 28 variables. These include two descriptive variables regarding the intervention, one of which details the Unique Project Code (CUP) associated with each intervention. Additionally, there is information on the title and description of the intervention, along with the rationale behind its necessity. Sometimes, specific location details such as the address of the works are also provided. A financial variable denotes the total investment amount, while six geographic variables capture region, province, and municipality codes and denominations. Sixteen variables track key dates in the public works process. There's one variable indicating the source of the data. Furthermore, two geo-reference variables are included; one indicating point coordinates, and the other delineating the polygonal shape of each municipality area (see Table 1). In the repository there is also a file called metadata where information regarding each variable can be found. The temporal expanse of the collected data is particularly notable, extending from the late 90’s to the current date. Specifically, there are relatively few observations up to the early 2000’s, attributed to the challenges of tracking interventions and a lack of robust data infrastructure. Municipalities exhibit varying spans of available information, reflecting the uneven temporal coverage of the dataset. This variability in temporal data coverage further complicates comprehensive analysis and monitoring of soil defence interventions across different regions. Additionally, the dataset can be considered complete and consistent starting from a specific year. Specifically, from 2000 onwards, the data can be regarded as consolidated for the entire national territory.

**Table 1:** Variables of the dataset.

Variable	Description	Format
CUP	Unique Project Code	Character
Intervention	Title and description of the Intervention	Character
Finance	Total amount of Investments financed for the Intervention	Numeric
COD_REGION	ISTAT Region Code	Character
DEN_REGION	Region Denomination	Character
COD_PROVINCE	ISTAT Province Code	Character
DEN_PROVINCE	Province Denomination	Character
COD_MUNICIPALITY	ISTAT Municipality Code	Character
DEN_MUNICIPALITY	Municipality Denomination	Character
FeasibilityStudyStartingDate	Starting Date of the Feasibility Study Phase	YYYY-MM-DD
FeasibilityStudyEndingDate	Ending Date of the Feasibility Study Phase	YYYY-MM-DD
PreliminaryDesignStartingDate	Starting Date of the Preliminary Design Phase	YYYY-MM-DD
PreliminaryDesignEndingDate	Ending Date of the Preliminary Design Phase	YYYY-MM-DD
DefinitiveDesignStartingDate	Starting Date of the Definitive Design Phase	YYYY-MM-DD
DefinitiveDesignEndingDate	Ending Date of the Definitive Design Phase	YYYY-MM-DD
ExecutiveDesignStartingDate	Starting Date of the Executive Phase	YYYY-MM-DD
ExecutiveDesignEndingDate	Ending Date of the Executive Phase	YYYY-MM-DD
EffectiveDesignStartingDate	Starting Date of the Effective Design Phase	YYYY-MM-DD
EffectiveDesignEndingDate	Ending Date of the Effective Design Phase	YYYY-MM-DD
WorksExecutionStartingDate	Starting Date of the Works' Execution	YYYY-MM-DD
WorksExecutionEndingDate	Ending Date of the Works' Execution	YYYY-MM-DD
ConclusionStartingDate	Starting Date of the Conclusion Phase	YYYY-MM-DD
ConclusionEndingDate	Ending Date of the Conclusion Phase	YYYY-MM-DD
InterventionClosed	Date of the Intervention Closing	YYYY-MM-DD
Operability	Date of the Operability of the Intervention	YYYY-MM-DD
Source	Source of the Data	Character
geom_C	Centroids of the Municipality	Point
geom_A	Areal shape of the Municipality	Polygon and Multi-polygon

(Fig. 1) shows some missing municipalities. In particular, there are 1699 municipalities which do not have any records. It is possible to see that the majority of these municipalities are very small, and mainly located in the hinterland. Several factors might explain why hinterland municipalities lack specific data on soil defence interventions. First, geographic and demographic characteristics might contribute to the absence of recorded information. Furthermore, limited resources and budgets, lower population densities, and accessibility issues can hinder the implementation and documentation of such measures. Additionally, there might be a lack of awareness or expertise regarding soil defence strategies in these areas. Also, it may be that economic priorities do not emphasize environmental interventions. Historical gaps in data collection and reporting infrastructure also contribute to the absence of information. The lack of data on soil defence interventions is crucial as it underscores potential gaps in environmental monitoring and the need for improved documentation and support for these regions.

## 4 Experimental Design, Materials and Methods

Three sources are used to retrieve the soil defense public works data: the OpenBDAP, the OpenCoesione, and the ReNDiS repositories. The Open BDAP [7] database is maintained by the Italian Ministry of Economics and Finance (MEF), specifically by the *Ragioneria Generale dello Stato.* Although it primarily houses data related to the whole Italian Public Administration, our interest specifically centers on the dataset concerning investments made for public works, with a particular emphasis on soil defense. This dataset, updated monthly, encompasses a range of information about the interventions, including their nature, project code, investment sectors, descriptions of the interventions, implementation timelines, sub-sector details, and other pertinent attributes. From a financial perspective, the dataset offers insights into the costs associated with interventions and distributes financial flows among national, European, local authorities, and private entities. This allocation of financial resources is presented at an aggregated level, distinguishing between sources such as European Fundings, National Fundings, contributions from Local Authorities, foreign countries or private entities. Moreover, the dataset covers data registered mainly from the year 2000 to the present day, showing the dates of the most meaningful steps of public works’ projects.

The second source is the OpenCoesione [8]. It draws its information from Cohesion Policy data, in which EU member states commit to delivering cohesive programs and data at both national and European levels. This resource is updated bi-monthly, offering a broad view of public investments sourced from both Italian and European funding channels.

Even in this case, the data collected are specific to single projects and are referenced at the municipal level revealing the various resilience measures undertaken by each municipality, along with the corresponding allocated budgets. This database offers a detailed view of the economic investments regarding all public infrastructures: it collects data from both national and European programs and provides a comprehensive breakdown of the financing allocation between national and European resources. Specifically, it delineates the total amount of financing and specifies the respective contributions from specific national and European funds. Also in this case, dates about the legislative process of public works are reported.

As previously mentioned, these two repositories gather data on various types of public works, including school infrastructure, transportation systems, and more. However, our analysis exclusively concentrates on soil defense interventions. To achieve this focus, the data has been filtered using the sub-sector code provided by both datasets. Specifically, the sub-sector code "05" signifies soil defense investments, enabling us to isolate relevant interventions.

Last repository is the 'Repertorio Nazionale degli Interventi per la Difesa del Suolo', (ReNDiS, [6]), provided by the Italian Institute for Environmental Protection and Research (ISPRA). It is a database entirely dedicated to public investments in soil defense. Currently, the ReNDiS presents data on the total amount of financial resources allocated to each project, the category of intervention, the project code, and key dates in the public works process based on the different phases reported by the Open BDAP repository. The primary goal of the ReNDiS platform is to create a comprehensive, systematically updated overview of the works and resources engaged in soil defense. While the MEF provides a more detailed and comprehensive database as the primary source, it's crucial to highlight that the implementation of the ReNDiS database is an ongoing process. Currently, it features information sourced from the Ministry of the Environment, and efforts are underway to incorporate details regarding interventions financed through additional channels, including Regional Laws, ordinances, and other funding sources.

Furthermore, data from the three different sources are collected using two different methods: for the OpenCoesione and the OpenBDAP Application Programming Interface (API) have been used to process and download the data, while the ReNDiS database is shared in a LinkedOpenData platform, thus it can be retrieved through SPARQL queries. Particularly, in the context of data retrieval, APIs provide structured access to external databases, enabling the extraction of specific data. Specific API requests were crafted to fetch relevant data [11]. These requests included parameters to filter the data regarding public investments for each region. On the other hand, SPARQL is a query language and protocol used for accessing and manipulating data stored in Resource Description Framework (RDF) format [12]. SPARQL is particularly useful for querying Linked Open Data (LOD), where data entities are interlinked and accessible on the web. Indeed, ISPRA is implementing its LOD platform sharing all the data it collects. In this particular case, the ReNDiS database is accessible via a SPARQL endpoint (https://dati.isprambiente.it/sparql), which acts as an interface for executing SPARQL queries.

The two retrieving methods were implemented in the RStudio IDE for the R software environment [9], where also the data processing was carried out.

Once the three databases are collected the main part is to merge them into a unique database without any duplication. For this purpose, each project is uniquely identified by a project code known as the *Codice Unico di Progetto* (CUP). The CUP system has played a pivotal role in monitoring and consolidating information related to public investments, as mandated by the *Sistema di Monitoraggio degli Investimenti Pubblici* (MIP), established under Law 144 in 1999. Furthermore, the adoption of the CUP system has been in effect since January 16, 2003, through Law No. 3.

The utilization of the CUP has served as a mechanism to test data duplication across the three datasets, ensuring the absence of redundant information. This systematic approach guarantees the integrity and accuracy of the dataset by confirming that no duplicate entries exist, thereby enhancing the reliability of the collected project data.

As introduced before, the three datasets also report key dates regarding the legislative process of public works that in Italy follows four different phases: Planning, Implementation, Conclusion, Operability. The Open Coesione database provides a more granular breakdown of project phases, furnishing both effective and expected dates and categorizing them into six distinct sub-phases. These sub-phases include the feasibility study, preliminary design, definitive design, executive design, execution of works, and testing. Each sub-phase is accompanied by its corresponding start and end dates.

In contrast, the OpenBDAP platform consolidates these phases into the four macro phases, consistently presenting both expected and effective dates, along with their respective start and end dates. The ReNDiS database draws its information from OpenBDAP, resulting in identical date records. To streamline the dataset and adopt a pragmatic approach, only effective dates are reported.

The OpenBDAP's metadata file elucidates the derivation of these dates, facilitating the merging of datasets, particularly it helps reduce the OpenCoesione dates to the four phases present in both OpenBDAP and ReNDiS. Specifically, the effective start date for the design phase is determined as the minimum effective date among the start dates of the feasibility study, preliminary design, definitive design, and executive design. Conversely, the end date aligns with the maximum effective date among the end dates of these aforementioned phases.

For the execution phase, the effective start date is derived as the minimum among the start date of works execution, while the end date considers the maximum end date of the phase. The start date of the conclusion phase is determined by the minimum start date among the testing and closing intervention phases, applying a similar approach to establish the end date for this phase.

Lastly, the start date of the operability phase corresponds to the minimum effective start date of the operability phase.

Consequently, the OpenCoesione database's date information has been condensed by identifying the minimum or maximum of start or end dates, ensuring alignment with the dates reported in the OpenBDAP and ReNDiS databases.

Moreover, the financial information from the three datasets is different. OpenCoesione shows a very detailed partitioning among European and Italian resources, showing different funds for both levels.

European funds refer to:

‐ European Regional Development Fund (ERDF)
‐ European Social Fund (ESF)
‐ European Agricultural Fund for Rural Development (EAFRD)
‐ European Maritime and Fisheries Fund (EMFF)
‐ Youth Employment Initiative (YEI)

On the other hand, the Italian funds are:

‐ Fondo di Rotazione nazionale
‐ Fondo Sviluppo e Coesione (FSC)
‐ Piano di Azione per la Coesione (PAC)

Additionally, the dataset also differentiates between investments originating from the Italian Government, Regions, Provinces, and Municipalities.

The OpenBDAP dataset also reports different fund allocations but in a more aggregated way. It distinguishes the European Resources and National Resources, which are given respectively by the sum of the European funds and National funds. Also, it presents the category of the Local authorities' funds, given by the sum of the Regions, Provinces, and Municipalities financing.

In contrast, the ReNDiS database does not have any differentiation of funds implemented, but it reports only the total amount financed for each intervention. For this reason, the database presents only one financial variable representing the total amount of financing per intervention. In the case of the OpenBDAP and the OpenCoesione datasets this information is derived as the sum of all the financial values reported in each fund's resources. To validate the accuracy of this sum, we meticulously scrutinized duplicated interventions and confirmed that the total amount reported by the ReNDiS database aligns precisely with the combined financial resources reported in the other two datasets.

A key and innovative point of the creation of this dataset is that the three databases have been enriched with geographical information. The OpenCoesione and the ReNDiS datasets present region, province and municipality codes and denominations derived from the official source of the Italian National Statistics Institute (ISTAT). On the contrary, the OpenBDAP dataset does not present this information in the financial dataset, but the same data platform provides these codes and denominations associated to the CUP of each project in a different database. The latter has been downloaded and the geographical information added to the financial dataset.

Thanks to this transformation for the three datasets two different geographical reference systems have been implemented: the coordinates of the centroids of the municipality of each intervention and the areal data outlining the polygonal shape of each municipality. These data options are sourced from the ISPRA database through queries, providing flexibility in accessing the geographical information of municipalities. In the ReNDiS database, georeferencing is seamlessly integrated during the data download via queries, leveraging the existing geo-references within the database. In the Open Coesione and OpenBDAP datasets, centroids or polygons are linked through province and municipal codes.

## Limitations

The dataset provides insights into soil defence investments in Italy, although it presents certain limitations. Firstly, it may not capture all soil defence initiatives carried out in Italy, leading to gaps in information and underrepresentation of certain regions or types of projects. Indeed, its reliance on publicly available sources may introduce biases in the data, particularly if certain projects or regions are underrepresented or omitted from the dataset. This is particularly relevant for the initial phase of the data retrieval, lasting up to around the early 2000’s.

The dataset's temporal coverage restrains the analysis of long-term trends and the assessment of the sustainability of soil defence efforts. Furthermore, it may lack detailed information on contextual factors such as socio-economic, or environmental influences, which could impact the depth of analysis and understanding of soil defence dynamics.

Additionally, while efforts were made to ensure data accuracy and completeness, reliance on administrative records and self-reporting mechanisms may introduce errors or inconsistencies. Therefore, users should exercise caution when interpreting the findings and consider conducting sensitivity analyses to assess the robustness of the results. Despite limitations, the dataset remains a valuable resource for researchers and policymakers, but cautious interpretation and additional contextual research are advised.

## Ethics Statement

The authors confirm that they have read the ethical requirements for publication in Data in Brief and confirm that the data presented do not involve human subjects, animal experiments, or any data collected from social media platforms.

As the data contained in the dataset are already part of the public domain under CC-BY Licence, no permission to use primary data was needed.

## CRediT authorship contribution statement

**Lorena Ricciotti:** Conceptualization, Software, Data curation, Writing – original draft. **Alessio Pollice:** Writing – review & editing, Supervision.

## Data Availability

March 20, 2024 (v2)DatasetOpen Geo-referenced Harmonized Financial Data on Soil Defense Public Works in Italy (Reference data) (Zenodo).
